# Auditory driving of the autonomic nervous system: Listening to theta-frequency binaural beats post-exercise increases parasympathetic activation and sympathetic withdrawal

**DOI:** 10.3389/fpsyg.2014.01248

**Published:** 2014-11-14

**Authors:** Patrick A. McConnell, Brett Froeliger, Eric L. Garland, Jeffrey C. Ives, Gary A. Sforzo

**Affiliations:** ^1^Department of Exercise and Sport Sciences, Ithaca CollegeIthaca, NY, USA; ^2^Department of Neurosciences, Medical University of South CarolinaCharleston, SC, USA; ^3^College of Social Work and Huntsman Cancer Institute, University of UtahSalt Lake City, UT, USA

**Keywords:** auditory driving, autonomic, binaural-beat, exercise, heart rate variability, relaxation

## Abstract

Binaural beats are an auditory illusion perceived when two or more pure tones of similar frequencies are presented dichotically through stereo headphones. Although this phenomenon is thought to facilitate state changes (e.g., relaxation), few empirical studies have reported on whether binaural beats produce changes in autonomic arousal. Therefore, the present study investigated the effects of binaural beating on autonomic dynamics [heart rate variability (HRV)] during post-exercise relaxation. Subjects (*n* = 21; 18–29 years old) participated in a double-blind, placebo-controlled study during which binaural beats and placebo were administered over two randomized and counterbalanced sessions (within-subjects repeated-measures design). At the onset of each visit, subjects exercised for 20-min; post-exercise, subjects listened to either binaural beats (‘wide-band’ theta-frequency binaural beats) or placebo (carrier tones) for 20-min while relaxing alone in a quiet, low-light environment. Dependent variables consisted of high-frequency (HF, reflecting parasympathetic activity), low-frequency (LF, reflecting sympathetic and parasympathetic activity), and LF/HF normalized powers, as well as self-reported relaxation. As compared to the placebo visit, the binaural-beat visit resulted in greater self-reported relaxation, increased parasympathetic activation and increased sympathetic withdrawal. By the end of the 20-min relaxation period there were no observable differences in HRV between binaural-beat and placebo visits, although binaural-beat associated HRV significantly predicted subsequent reported relaxation. Findings suggest that listening to binaural beats may exert an acute influence on both LF and HF components of HRV and may increase subjective feelings of relaxation.

## INTRODUCTION

Binaural beating is an auditory illusion that is perceived when two or more pure-tone sine waves of similar but different frequencies (under 1500 Hz and less than 40 Hz apart) are presented dichotically via stereo headphones (Draganova et al., 2008). For example, if a 510 Hz pure tone is presented to a listener’s right ear while a 500 Hz pure tone is presented to the listener’s left ear, the listener perceives an illusory binaural beat with a frequency (perceived tempo) of 10 Hz. Binaural-beat perception originates in the brainstem’s inferior colliculi (Smith et al., 1975) and superior olivary nuclei (Oster, 1973), where sound signals from each ear are integrated, and continues as the neural impulses travel through the reticular formation up the midbrain to the thalamus (Swann et al., 1982), auditory cortices and other cortical regions (Draganova et al., 2008).

Research findings suggest that music and sound can modulate autonomic arousal through entrainment (Trost and Vuilleumier, 2013; Regaçone et al., 2014). Entrainment is a process through which two autonomous rhythmic oscillators with similar but different fundamental frequencies interact, resonate, and synchronize (Cvetkovic et al., 2009). Classic examples of entrainment include the synchronizing of human sleep-wake cycles to the 24-h cycle of light and dark (Clayton et al., 2005), the synchronization of a heartbeat to a cardiac pacemaker (Cvetkovic et al., 2009), and the use of rhythmic auditory stimulation in the rehabilitation of motor functions (Thaut and Abiru, 2010).

Numerous studies have reported positive effects of purported binaural-beat entrainment on clinically relevant outcomes including: heart rate, blood pressure, electrodermal response, and finger temperature (Kennerly, 2004), performance vigilance and mood (Lane et al., 1998), hypnotic susceptibility (Brady and Stevens, 2000), mental and physical relaxation (Foster, 1990), attention and memory (Kennerly, 1994), depression and mood regulation (Cantor and Stevens, 2009), generalized anxiety (Le Scouarnec et al., 2001), as well as pre-operative anxiety and intra-operative anesthesia requirements (Kliempt et al., 1999; Lewis et al., 2004; Padmanabhan et al., 2005; Dabu-Bondoc et al., 2010). Many of these studies employed the Hemi-Sync^®^ auditory-guidance system (which combines binaural beats, music, pink noise, natural surf sounds, and verbal guidance) which is designed to employ ‘brainwave entrainment’ and facilitate ergotropic (increasing arousal) or trophotropic (decreasing arousal) changes in consciousness (Atwater, 2004). In spite of these prior positive findings, it remains uncertain whether binaural beats alone modulate autonomic arousal. In order to ascertain the clinical effectiveness of binaural beats, they must be experimentally isolated from possible confounding variables such as verbal guidance and instrumental music.

In the present study, we chose to employ theta-frequency (4–7 Hz) binaural beats to facilitate the post-exercise relaxation response. The relaxation response is an innate physiological response characterized by diminished sympathetic nervous system (SNS) activity and increased theta-brainwave activity (Benson et al., 1981). Interestingly, it has recently been shown that combining exercise—a practice known to produce anxiolytic effects (Raglin and Morgan, 1987) and improve long-term stress-resiliency (Salmon, 2001)—with subsequent relaxation training significantly reduced blood pressure and post-exercise blood pressure response to a laboratory stressor (Santaella et al., 2006). Therefore, exercise followed by conscious relaxation may provide for a deeper relaxation response than either intervention alone – a finding that might inform treatment for a wide-variety of stress-related conditions. In addition to the aforementioned positive effects of combined exercise and relaxation training, the decision to investigate binaural-beat effects post-exercise was made in an effort to capitalize on known autonomic effects of exercise and exercise-recovery (Parekh and Lee, 2005).

Briefly, exercise serves as an ergotropic stimulus which increases SNS activity (Bricout et al., 2010). In healthy populations, exercise elicits characteristic intensity- and duration-dependent effects which can interact with fitness level (i.e., VO_2_max; Buchheit and Gindre, 2006). We aimed to induce sympathetic activation via exercise, and then compare the effects of binaural beats to those of a placebo on post-exercise autonomic arousal, as indicated by heart rate variability (HRV) – a sensitive probe of autonomic tone. HRV was chosen as an autonomic probe (opposed to other measures such as event-related potentials or skin conductance) due to the monitor’s low-cost, minimal invasiveness, and portability. Generally, the effects of exercise include increased low-frequency (LF) power [a measure of both parasympathetic (PNS) and sympathetic (SNS) activity] and decreased high-frequency (HF) power (reflecting PNS activity) relative to pre-exercise values, with the net effect of increasing sympathetic dominance (i.e., LF/HF ratio; Parekh and Lee, 2005). Recovery from moderate/intense exercise normally involves an acute reduction in LF power and an increase in HF power, which then typically return to near baseline levels within 30-min to an hour – resulting in the eventual restoration of baseline sympathovagal balance (Terziotti et al., 2001; Gladwell et al., 2010). It is important to note that while exercise-induced increases in SNS activity can be inferred through the LF component of HRV, LF HRV signal is contributed to by both SNS and PNS components – making interpretations based on LF power alone somewhat dubious (Camm et al., 1996). HF HRV signal, however, is considered to be exclusively mediated by PNS. Post-exercise, heart rate decreases towards baseline levels, reputedly through a combination of SNS withdrawal and increased PNS activation (Pierpont and Voth, 2004). For heart rates above 100 bpm, SNS withdrawal dominates; as heart rate falls below 100 bpm, further reductions are primarily mediated by PNS activation (Pierpont and Voth, 2004).

First, we hypothesized that exercise would decrease parasympathetic activity (as measured through decreased HF HRV component) and increase sympathetic activity (as measured through increases in the LF HRV component). Second, we hypothesized that exposure to theta-frequency binaural beats (relative to placebo) would result in increased parasympathetic activity following exercise (increased HF HRV component). Third, we also hypothesized concomitant decreases in the LF HRV component (reflecting a combination of parasympathetic and sympathetic activity), as well as in overall LF/HF ratio – often referred to as sympathovagal balance. Lastly, we hypothesized that binaural beats would facilitate entry into a deeper state of relaxation, with participants reporting increased perceived relaxation during the binaural-beat condition relative to the placebo condition.

## MATERIALS AND METHODS

### SUBJECTS

Twenty-two college students were recruited by announcement and signed informed consent approved by Ithaca College’s institutional research review board. Subjects remained naïve to the true nature of the experiment; they were told only that the study was designed to examine the effects of music on exercise recovery. Subjects’ health-histories were assessed; exclusion criteria included high cardiovascular risk, habitual smoking, chronic alcohol usage, prescription medication usage or a history of diagnosed mental or physical illness. One subject’s data were excluded from analyses due to failure to complete both sessions, resulting in an *n* of 21 for final analyses. Eleven subjects received placebo condition first and ten subjects received binaural-beat condition first; no significant differences between assignment groups for any variable were observed (*p* > 0.05). Baseline and post-exercise descriptive statistics for subjects are found in **Table 1**.

**Table 1:** Descriptive statistics for subjects’ baseline and post-exercise data.

	Mean (*M*)	Standard Deviation (*SD*)
Age	20.33	2.69
Height (cm)	171.12	10.24
Weight (kg)	77.41	15.31
Body mass index	26.1	4.1
VO_2_max (mL/kg/min)	48.3	6.5
ExRx-MPH	5.8	0.8
Sex (no.)	14 male/7 female	
**Ethnicity (no.)**
White/Caucasian	16	
Black/African American	2	
Asian	1	
Hispanic/Latino(a)	1	
Unreported	1	

*VO_2_max = maximal *predicted* volume of oxygen consumption; ExRx-MPH = exercise prescription in miles per hour.*

#### Cardiovascular measures and analyses

A Polar RS800CX heart rate monitor (Polar Electro Oy, Kempele, Finland) was used to record heart rate, as it has been previously demonstrated to provide reliable measures to calculate HRV (Wallén et al., 2011) – an important indicator of relaxation (Peng et al., 2004) and sympathovagal balance (Sharpley et al., 2000). Subjects’ R–R interval data were sampled at a 1000 Hz sampling frequency, allowing for a 1-ms temporal resolution of HRV (Polar RS800CX User Manual, 2011). Heart rate data for each subject were uploaded to Polar ProTrainer 5 software (Version 5.40.172).

#### Acoustic equipment

A sound level meter (Model 33-2050, Radio Shack, Tandy Corporation, Fort Worth, TX, USA) was used with a C-weighted slow-response setting to calibrate a pair of Koss HQ1 collapsible full-size headphones (Milwaukee, WI, USA), and to standardize volume levels. Volume levels for both left and right earpieces, and for both placebo and binaural-beat audio tracks, ranged from 61 to 63 decibels (dB).

During the experimental session, multiple carrier tones were presented over a background of pink noise (20–20,000 Hz with power attenuated in non-audible frequency ranges) with a 7 Hz interaural frequency difference that was continuously varied by plus or minus 1.5 Hz over a 4-s period (i.e., looped from 7 to 8.5 to 7 to 5.5 Hz and back; The Monroe Institute, Faber, VA, USA). Over the course of a 20-min presentation, carrier tones were changed in seamless ten-second cross fades to facilitate listener vigilance. This ‘wide-band’ binaural-beat effect was created through the presentation of the following carrier tone chords: zero to 3-min, C-Major-seventh; 3-min to 5-min, C-Major; 6-min to 10-min, G-Major; 10-min to 15-min, D-Minor; and 15-min to 20-min, C-Major (Brady and Stevens, 2000). The audio tracks (placebo vs. binaural-beat) were designed to be perceptually indistinguishable from one another in naïve listeners. Both audio tracks were provided by The Monroe Institute, Faber, VA, USA (Atwater, unpublished manuscript). During the placebo session, subjects listened to pink noise with identical carrier tones as in the binaural-beat condition but with no varying interaural phase difference.

#### Perceived relaxation scales

At the end of each experimental session, subjects were asked to rate the degree of their perceived relaxation during the relaxation protocol on a scale of 1–10, with one indicating the least relaxed and 10 indicating the most relaxed. Single-item numeric rating scales have been previously used in the literature to measure subjective responses to music (Iwanaga et al., 2005; Tan et al., 2012) as well as to other interventions (Strauser, 1997).

### PROCEDURES

**FIGURE 1:**
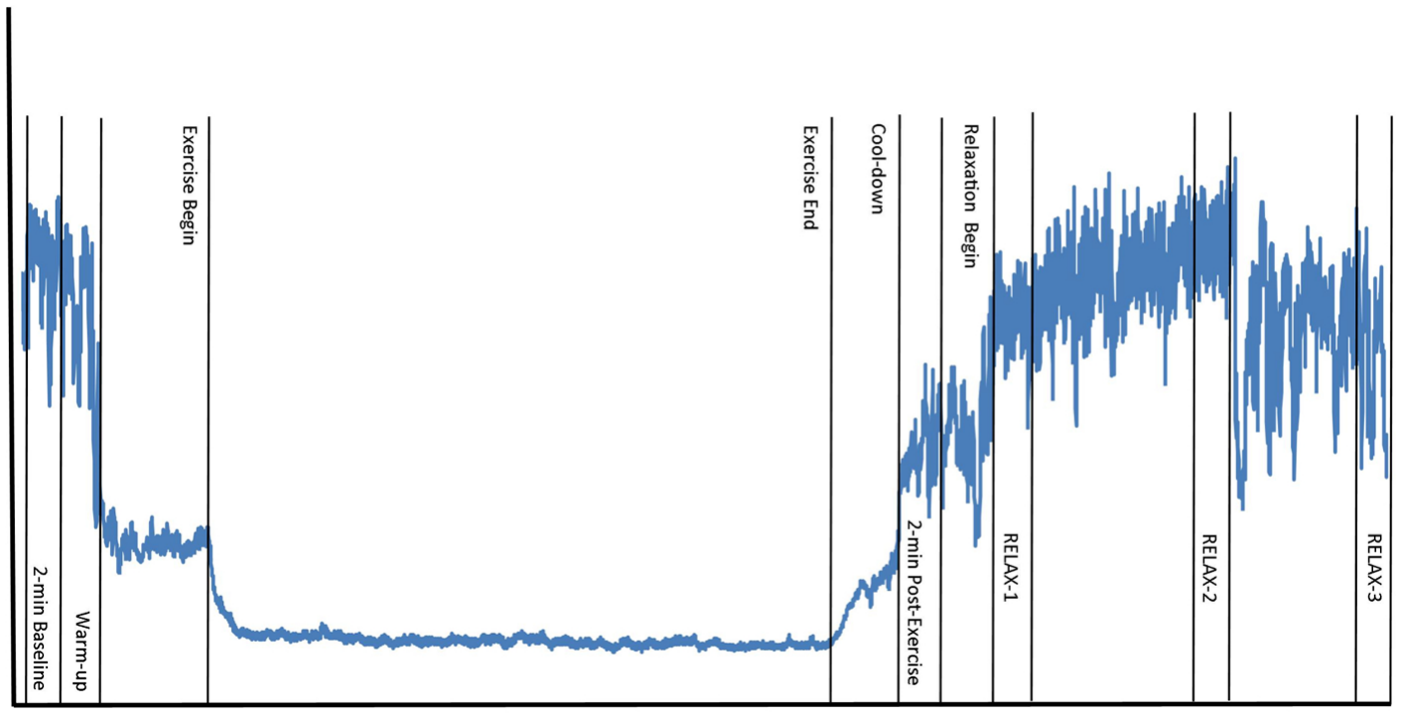
**Testing schedule and data collection time points.** Wavy line represents example R–R interval time series over the course of one experimental session, including the following time points: 2-min baseline, 5-min warm-up, 20-min exercise, 5-min cool-down, 2-min post-exercise, 20-min relaxation [heart rate variability sampled during 2-min windows at beginning (RELAX-1), middle (RELAX-2), and last 2-min (RELAX-3) of relaxation].

Prior to each experimental session, subjects were emailed instructions to avoid strenuous exercise, alcohol, and over-the-counter medication for 24-h prior to the session, and caffeine within 3-h of the session. A 24-h history questionnaire was administered to each subject at the beginning of each experimental session to assess compliance with study instructions. Participants who failed to comply with all study requirements over the 24-h period prior to testing were excluded from participation (one subject due to medical reasons). To control for diurnal hormone fluctuations known to play a role in HRV, each subject was scheduled at the same time of day for each experimental session (Armstrong et al., 2011). Subjects were alternately assigned to A–B and B–A conditions to control for order effects and the researcher conducting the experiment was blinded to condition. Subjects returned within a 2-week period to complete the alternate condition. On subjects’ first visit, height and weight were measured. Subjects were seated and instructed to complete paperwork required for a predicted VO_2_max regression formula (Bradshaw et al., 2005). After completion, they were told to sit quietly and relax for 5-min. An exercise protocol was individually determined as per American College of Sports Medicine guidelines for moderate cardiovascular exercise (70% of predicted VO_2_max; Thompson, 2010) with a 5-min warm-up and cool-down at 50% of prescribed workload. Subjects performed treadmill (Precor 956; Woodinville, WA, USA) exercise in order to elicit a strong sympathetic nervous system response. Heart rate was recorded continuously throughout the experiment. HRV was sampled during 2-min windows of quiet rest while in an upright position: at baseline, post-exercise, and at the beginning (RELAX-1), middle (RELAX-2), and end (RELAX-3) of the relaxation protocol (**Figure 1**).

After completion of post-exercise measurements, subjects were instructed to sit in a recliner, lights were dimmed, a curtain was drawn around the recliner, and the following short script was read:

“This relaxation period will last 20-min. I will close this curtain and leave the room so you can relax and have the space to yourself. I will return after 20-min and open the curtain and we will move back over to the other chair. Once you put on the headphones and begin listening to the music, I need you to close your eyes, focus attentively on the music and relax as deeply as you can. Do you have any questions?”

Subjects attended two laboratory sessions: once while listening to binaural beats, once while listening to a placebo (carrier tones and pink noise only). Following 20-min of stimulus exposure via stereo headphones at a standardized volume, subjects were asked to rate their perceived degree of relaxation during the relaxation protocol. After completion of the study, subjects were debriefed (i.e., provided with information about binaural-beats).

### DATA PROCESSING

All heart rate signal time series were inspected for artifacts usingPolar ProTrainer to ensure that no signal contained more than 2% artifacts (Camm et al., 1996; Bricout et al., 2010). Heart rate R–R interval data were subsequently processed in Kubios HRV (Version 2.1; Tarvainen and Niskanen, 2008). Time series for all subjects were detrended using a smoothness priors based detrending approach with λ = 500 (smoothing parameter with cut-off of 0.010 Hz times the sampling frequency), f_c_ = 0.035 Hz (estimated cut-off frequency of the filter). Detrending removes slow-trend (Schmidt et al., 2010), and non-linear trend (Manimmanakorn et al., 2011), components which can cause distortion in the signal. Next, a conservative interpolation artifact correction algorithm was employed, using a ‘medium’ level of correction which excluded all obvious artifacts from analysis (Tarvainen and Niskanen, 2008). No more than 0.78% of R–R time-series (32/4069 beats) were interpolated. Frequency-domain HRV indices were calculated using a Fast-Fourier Transform (FFT) based Welch’s Periodogram method (Tarvainen and Niskanen, 2008), with a 256-s window width and a 50% window overlap. A standard setting with a 4 Hz interpolation rate was used with the following frequency bands: very-low frequency (VLF, 0–0.04 Hz), LF (0.04–0.15 Hz), and HF (0.15–1.0 Hz; Tarvainen and Niskanen, 2008). Consistent with prior exercise research, the extended HF band was included for analysis as so to include frequencies resulting from post-exercise tachypnea which might otherwise be missed (Brenner et al., 1998; Sumi et al., 2006). After detrending and applying artifact correction, each signal was cut into five 2-min samples (baseline, post-exercise, beginning, middle, and end of relaxation protocol) providing adequate duration to assess short-term spectral components (Camm et al., 1996).

### DATA ANALYSIS

#### Preliminary analyses

First, all HRV variables were natural-log transformed to fulfill normality assumptions of parametric statistical testing. All data were then reverse-log transformed prior to reporting. Cohen’s *d*, a non-biased measure of effect size, was calculated for significant within-subjects results based on a correction for dependent means (Morris and DeShon, 2002; Wiseheart, 2013). For significant interactions, η^2^ is reported as an indicator of effect size. All analyses were two-tailed with α = 0.05.

Repeated-measures ANCOVA were performed for all baseline and post-exercise measures of HRV to test for differences between conditions in pre-relaxation HRV values (**Table 2**). In an effort to isolate potential binaural-beat treatment effects from any confounding effect of exercise, delta scores for each session (2-min immediately post-exercise minus the 2-min baseline) were computed and included as nuisance covariates in all HRV analyses – the intended effect being to explain known variance in HRV at the onset of relaxation resulting from baseline and post-exercise differences in HRV between conditions. In order to control for known differences in vagal mediation of cardiac control due to aerobic fitness level, sex/gender, and age (Stein et al., 1997; Rossy and Thayer, 1998), VO_2_max, sex, and age were included as nuisance covariates in all HRV analyses.

**Table 2:** Baseline and post-exercise measures of heart rate variability.

	Placebo *M* (*SE*)	Binaural-beat *M* (*SE*)	*F (p)*
Baseline heart rate	71.88 (1.03)	69.55 (1.03)	0.49 (0.492)
Baseline LF power	66.62 (1.05)	57.86 (1.05)	4.00 (0.063)
Baseline HF power	27.61 (1.11)	31.41 (1.07)	4.43 (0.051)
Baseline LF/HF ratio	2.42 (1.17)	1.84 (1.11)	5.25 (0.035)*
Post-Ex heart rate	98.00 (1.04)	97.61 (1.03)	0.02 (0.884)
Post-Ex LF power	82.76 (1.03)	82.76 (1.03)	1.34 (0.255)
Post-Ex HF power	13.64 (1.16)	14.00 (1.15)	1.10 (0.310)
Post-Ex LF/HF ratio	6.07 (1.19)	5.91 (1.18)	1.17 (0.295)

All power measures expressed in normalized units. Age, sex, and VO_2_max included as covariates in model. *p< 0.05.

Given that the sample mean body mass index (BMI) was 26.1 (slightly overweight), additional correlational and ANCOVA analyses were performed to rule out a potentially confounding effect of BMI. To rule out a potentially confounding effect of condition order, this variable was included in all ANCOVA models as a between-subjects factor *post hoc*. When condition order was included in the ANCOVA models as a between-subjects factor, in each case significant findings became more significant and Condition × Time error was moderately reduced. No significant Condition × Time × Condition Order interactions were observed.

#### Primary analyses: Heart rate variability

To assess whether theta-frequency binaural-beats significantly altered sympathovagal balance (i.e., LF to HF normalized power ratio; LF/HF) over the course of the relaxation session (i.e., beginning, middle, and end), a 2 (Condition) × 3 (Time) repeated-measures analysis of covariance (RM-ANCOVA) was employed. Normalized LF and HF components were then assessed independently via 2 × 3 RM-ANCOVAs with the same covariates. Significant Condition × Time interactions were followed up with planned within-subjects repeated-measures contrasts (RELAX-1 to RELAX-2 and RELAX-2 to RELAX-3). To further characterize the nature of observed Condition × Time interactions, simple main effects of Time were assessed via within-subjects *F*-test within each condition separately; where significant *F-*statistics were observed, planned within-subjects contrasts were reported. Simple main effects of Condition were assessed at each time point, again via RM-ANCOVA. When observed, significant covariate interactions were explored by plotting said covariate against each condition’s delta regressor (i.e., RELAX-3–RELAX-1). In order to rule out gross patterns of autonomic difference, mean heart rate was also evaluated at baseline, post-exercise, and at each relaxation time point using RM-ANCOVA. Given the short duration of HRV recording samples, time-domain, and non-linear measures were not explored.

#### Secondary analyses: Perceived relaxation

Perceived relaxation ratings were assessed through paired-samples *t*-test. Effects of baseline variability in HRV on perceived relaxation were assessed using RM-ANCOVA; marginal means for significant results were reported. Relationships between in-session HRV measures and perceived relaxation were explored using bivariate correlation, partial correlation and linear regression.

## RESULTS

### BASELINE AND POST-EXERCISE MEASURES OF HEART RATE VARIABILITY

After controlling for age, sex, and VO_2_max, a significant difference was observed between conditions for baseline LF/HF ratio, with subjects exhibiting reduced baseline sympathovagal balance during the binaural-beat session (**Table 2**). No differences were observed between conditions for post-exercise HRV values, even after including baseline HRV values as covariates. Mean heart rate did not differ between conditions at any time point and was not significantly higher than baseline at RELAX-3 (all *p* > 0.05). Exercise significantly decreased HF power and increased LF and LF/HF powers during both conditions (all *p* < 0.05).

Body mass index was not correlated with any measure of HRV (i.e., HF, LF, or LF/HF components) during the relaxation protocol during either experimental condition (all *p* > 0.05). This remained the case after running partial correlations controlling for age, sex, and VO_2_max. To assess group differences while maintaining statistical power, participants were categorically coded in to low (BMI ≤ 25; *n* = 10), high (BMI ≤ 30; *n* = 8), and very high (BMI > 30; *n* = 3). This categorical variable, ‘BMI-level,’ was then added into the general linear model for each ANCOVA as a between-subjects variable. For HF, LF, and LF/HF, within-subjects ANCOVA results still showed a significant Condition × Time effect (all *p* < 0.05) with no significant Condition × Time × BMI-level interactions (all *p* > 0.05).

### EFFECTS OF BINAURAL-BEATS ON HEART RATE VARIABILITY

The effects of binaural-beats were assessed through three independent 2 × 3 RM-ANCOVAs for LF/HF, LF, and HF. Marginal means for HRV measures at the beginning (RELAX-1), middle (RELAX-2), and end (RELAX-3) of the relaxation protocol are shown in **Table 3**.

**Table 3:** Marginal heart rate variability means at onset, middle, and end of relaxation protocol.

	Placebo *M* (*SE*)	Binaural-beat *M* (*SE*)	*F* (*p*)
**Onset of relaxation**			
RELAX-1 Heart Rate	85.03 (1.03)	84.35 (1.03)	0.03 (0.873)
RELAX-1 LF Power	66.89 (1.06)	61.62 (1.07)	7.93 (0.013)*
RELAX-1 HF Power	27.91 (1.11)	28.56 (1.14)	2.44 (0.139)
RELAX-1 LF/HF Ratio	2.4 (1.17)	2.16 (1.21)	4.03 (0.063)
**Middle of relaxation**			
RELAX-2 Heart Rate	77.56 (1.03)	77.09 (1.04)	0.00 (0.961)
RELAX-2 LF Power	65.04 (1.07)	61.37 (1.07)	0.095 (0.762)
RELAX-2 HF Power	26.41 (1.14)	30.17 (1.13)	0.678 (0.423)
RELAX-2 LF/HF Ratio	2.46 (1.22)	2.03 (1.19)	0.543 (0.473)
**End of relaxation**			
RELAX-3 Heart Rate	75.04 (1.03)	73.70 (1.04)	0.03 (0.858)
RELAX-3 LF Power	59.2 (1.07)	59.15 (1.07)	1.33 (0.267)
RELAX-3 HF Power	34.47 (1.12)	32.95 (1.11)	2.10 (0.169)
RELAX-3 LF/HF Ratio	1.72 (1.20)	1.8 (1.17)	2.03 (0.174)

All power measures expressed in normalized units. Age, sex, VO_2_max, and ΔHRV (post-exercise – baseline; for each respective HRV variable) are included as covariates in the model. *p< 0.05.

#### Low-frequency to high-frequency ratio

**FIGURE 2:**
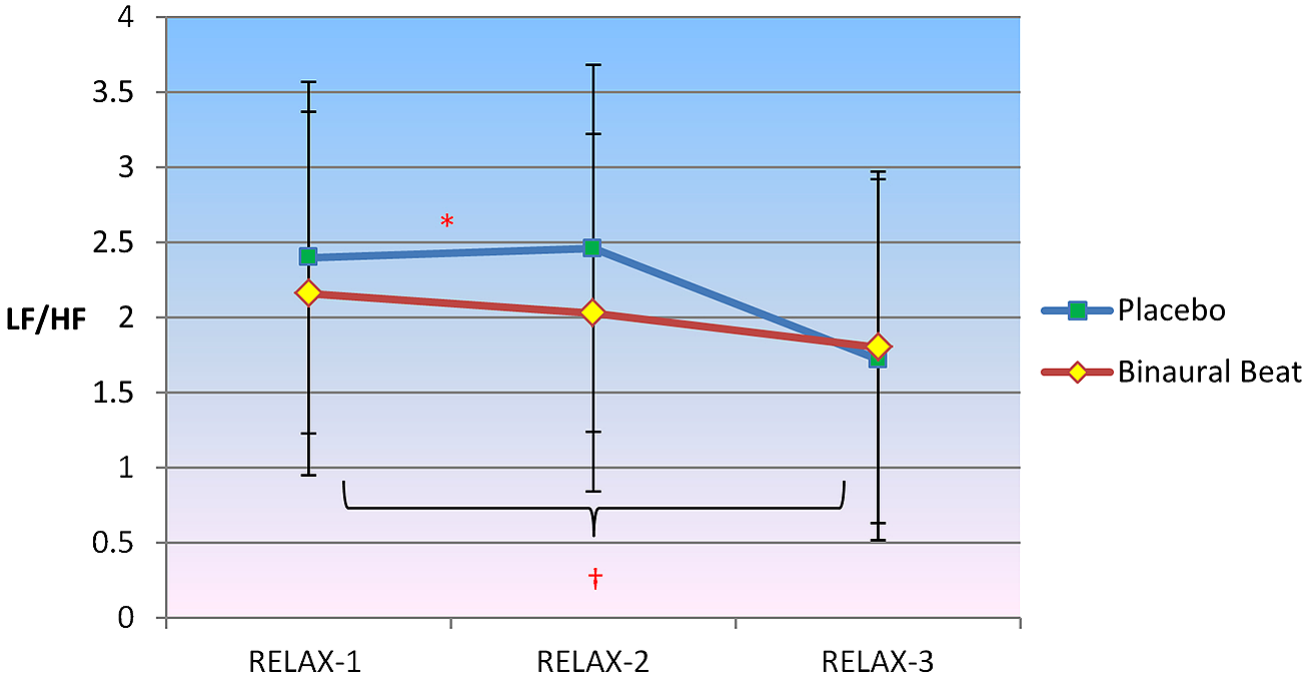
**Differences in sympathovagal balance (i.e., LF/HF) between binaural-beat and placebo conditions.** Error bars represent ±1 SE of the marginal mean. *Condition × Time interaction is significant at *p* < 0.05. ^†^Simple main effect of Time is significant at *p* < 0.05 (for binaural-beat condition only). *Interaction is significant at *p* < 0.05.

2 × 3 RM ANCOVA revealed a significant Condition × Time interaction for LF/HF, *F*(2,30) = 5.130, *p* = 0.012, η^2^ = 0.255 (**Figure 2**). LF/HF ratio increased in the placebo condition but decreased in the binaural-beat condition from the beginning to the middle of the relaxation protocol (i.e., from RELAX-1 to RELAX-2), *F*(1,15) = 5.427, *p* = 0.044, η^2^ = 0.245. No significant interactions between conditions were observed from the middle to the end of the relaxation protocol. There was a significant Condition × Time × VO_2_max interaction observed for LF/HF, *F*(2,30) = 3.793, *p* = 0.034, η^2^ = 0.202. However, the interaction of Time × VO_2_max was not significant in either condition (*p* > 0.05). When VO_2_max was regressed against delta (RELAX-3–RELAX-2) for the binaural-beat condition, β = -0.268; in the placebo condition, β = 0.139.

Follow-up simple main effects of Condition RM-ANCOVAs showed no significant difference between conditions for any time point (all *p* > 0.05; see **Table 3** above). No simple main effect of Time was observed for the placebo condition (*p* = 0.270); however a simple main effect of Time was observed in the binaural-beat condition [*F*(2,32) = 3.866, *p* = 0.031, η^2^ = 0.195]. No within-subjects contrasts were significant in the binaural-beat condition (all *p* > 0.05).

#### Low-frequency normalized power

**FIGURE 3:**
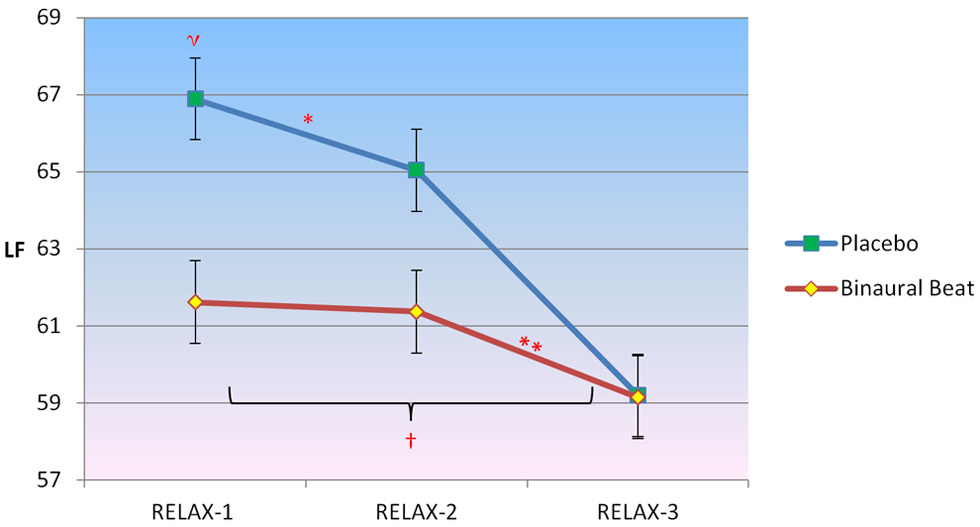
**Differences in the trajectory of low-frequency (LF) power (representing a combination of sympathetic and parasympathetic influences) over the course of relaxation between binaural-beat and placebo conditions.** Error bars represent ±1 SE of the marginal mean. *Condition × Time interaction is significant at *p*< 0.05. ^η^Marginal means are significantly different at *p*< 0.05. ^†^Simple main effect of time is significant at *p* < 0.05 (for binaural-beat condition only). **Within-subjects contrast from RELAX-2 to RELAX-3 is significant at *p*< 0.05.

2 × 3 RM ANCOVA showed a significant Condition × Time interaction for LF, *F*(2,30) = 7.202, *p* = 0.003, η^2^ = 0.324 (**Figure 3**). LF/HF ratio increased in the placebo condition but decreased in the binaural-beat condition from the beginning to the middle of the relaxation protocol (i.e., after 10-min of stimulus exposure), *F*(1,15) = 5.427, *p* = 0.044, η^2^ = 0.245. No significant interactions between conditions were observed from the middle to the end of the relaxation protocol. There was a significant Condition × Time × VO_2_max interaction observed for LF, *F*(2,30) = 4.806, *p* = 0.015, η^2^ = 0.243. The interaction of Time × VO_2_max was not significant in either condition (*p* > 0.05). When VO_2_max was regressed against delta (RELAX-3–RELAX-2) for the binaural-beat condition, β = -0.267; in the placebo condition, β = 0.272.

Follow-up simple main effects of Condition RM-ANCOVAs showed no significant difference between conditions for any time point (all *p* > 0.05; see **Table 3** above). No simple main effect of Time was observed for the placebo condition (*p* = 0.183), however, a simple main effect of Time was observed in the binaural-beat condition [*F*(2,32) = 4.057, *p* = 0.027, η^2^ = 0.202]. Within-subjects contrasts were significant in the binaural-beat condition from RELAX-2 to RELAX-3.

#### High-frequency normalized power

**FIGURE 4:**
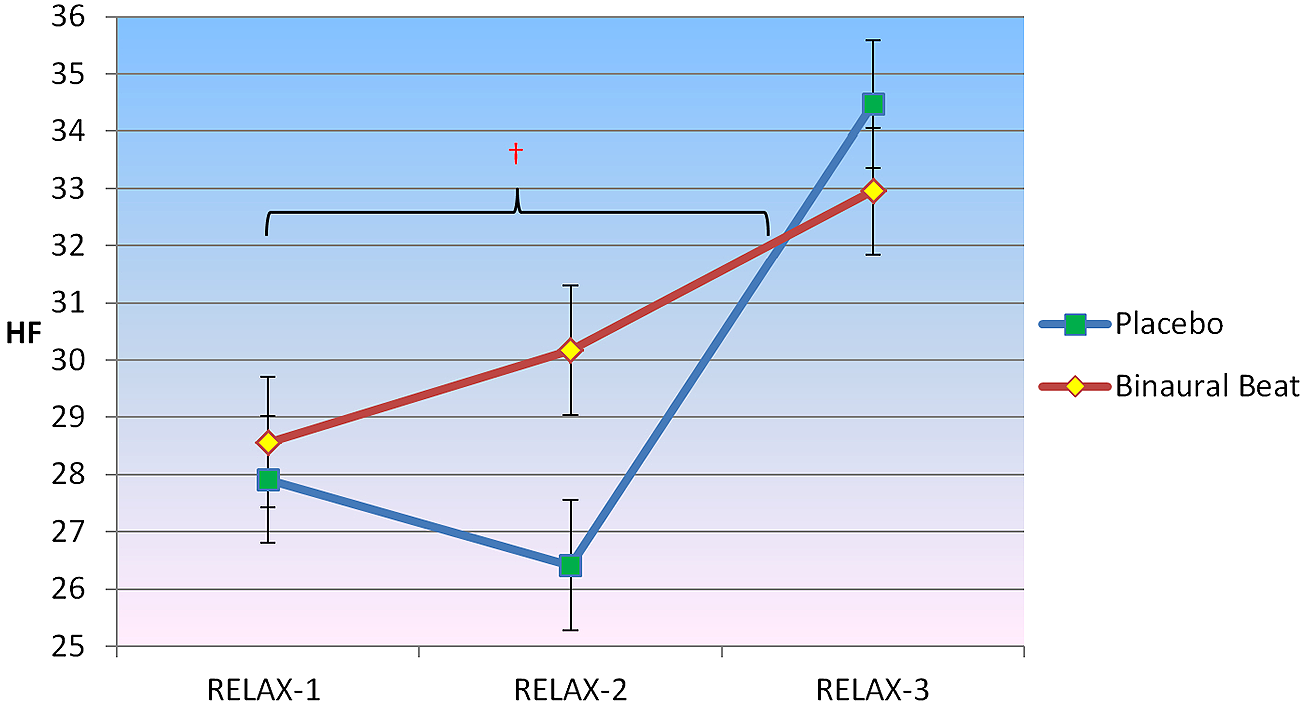
**Differences in the trajectory of high-frequency (HF) power (representing parasympathetic influence) over the course of relaxation between binaural-beat and placebo conditions.** Error bars represent ±1 SE of the marginal mean. ^†^Simple main effect of Time significant at *p* < 0.05 (for binaural-beat condition only).

2 × 3 RM ANCOVA showed a significant Condition × Time interaction for HF, *F*(2,30) = 3.811, *p* = 0.034, η^2^ = 0.203 (**Figure 4**). Planned follow-up within-subjects contrasts indicated an approach towards significance for HF, *F*(1,15) = 3.305, *p* = 0.089, from the beginning to the middle of the relaxation protocol. No significant interactions between conditions were observed from the middle to the end of the relaxation protocol. No Condition × Time × Covariate interactions were observed (*p* > 0.05).

Follow-up simple main effects of Condition RM-ANCOVAs showed no significant difference between conditions for any time point (all *p* > 0.05; see **Table 3** above). No simple main effect of Time was observed for the placebo condition (*p* = 0.295); however, a simple main effect of Time was observed in the binaural-beat condition [*F*(2,32) = 3.269, *p* = 0.051, η^2^ = 0.170]. No within-subjects contrasts were significant in the binaural-beat condition (all *p* > 0.05).

### EFFECTS OF BINAURAL BEATS ON PERCEIVED RELAXATION

**FIGURE 5:**
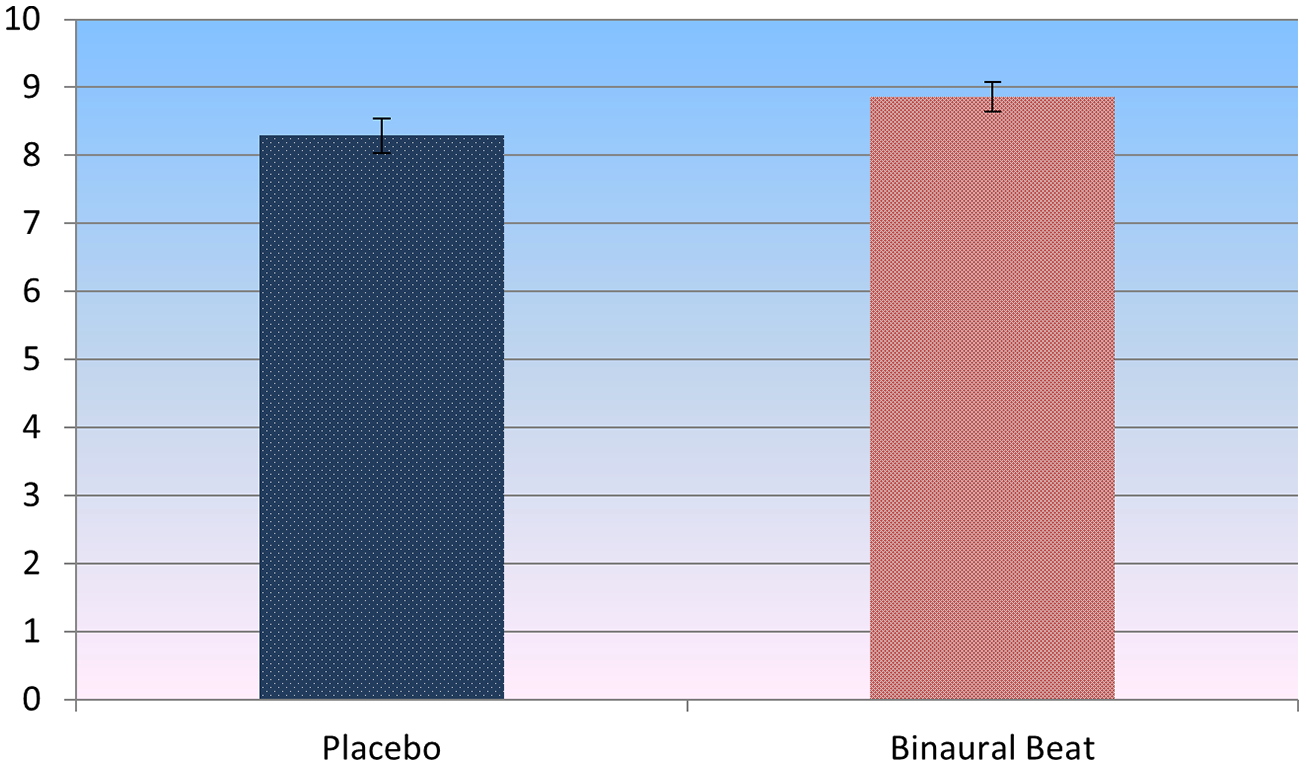
**Self-report ratings of perceived relaxation post-treatment (*t*= 2.248, *p* = 0.036, *d* = 0.493).** Error bars represent ±1 SE of the marginal mean.

Paired-samples *t-*test of post-treatment self-reported relaxation ratings revealed that subjects reported significantly more relaxation in the binaural-beat condition relative to the placebo condition (*p* = 0.036, *d*= 0.493; **Figure 5**). Importantly, this difference remained significant after including baseline LF/HF ratios as covariates in a RM-ANVOCA [*F*(1,18) = 5.75, *p* = 0.027, η^2^ = 0.242]. This provides some support for the idea that differences in self-reported relaxation were driven by binaural-beat exposure, not by baseline differences in autonomic tone.

### RELATIONS BETWEEN PERCEIVED RELAXATION AND HEART RATE VARIABILITY

**FIGURE 6:**
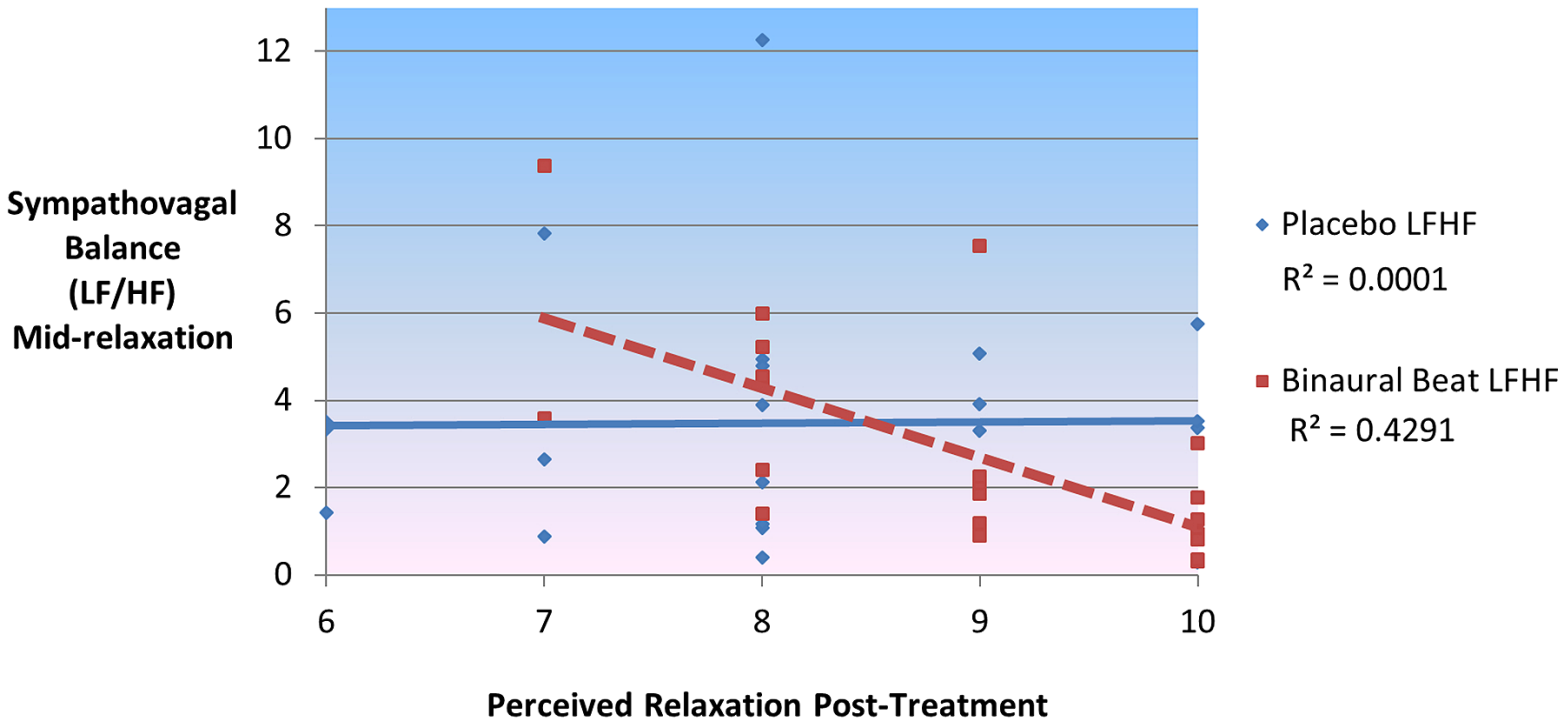
**Sympathovagal balance (LF/HF) mid-relaxation predicts 43% of variance in self-reported ratings of perceived relaxation in binaural-beat but not placebo condition**.

In the binaural-beat condition (but not in the placebo condition), LF/HF during the middle of relaxation was negatively correlated with, (*r*= -0.695, *p*< 0.001), and significantly predictive of, [*F*(1,20) = 17.786, *p*< 0.001], reported relaxation at the end of the session (**Figure 6**). This was the case for LF, (*r*= -0.640, *p* = 0.002; *F*(1,20) = 13.204, *p* = 0.002) as well; whereas HF was positively correlated with (*r*= 0.699, *p* < 0.001), and predictive of [*F*(1,20) = 18.203, *p* < 0.001], relaxation at the end of the session. To rule out the possibility that baseline LF/HF was responsible for the observed differences in perceived relaxation, a regression model was performed for each condition between baseline LF/HF, RELAX-2 LF/HF, and perceived relaxation. While both conditions’ baseline LF/HF values were significantly predictive of LF/HF mid-relaxation [*F*(1,20) = 8.409, *p* = 0.006 and *F*(1,20) = 12.566, *p* = 0.002, respectively], neither conditions’ baseline LF/HF values were significantly predictive of self-reported perceived relaxation at the end of the study (all *p* > 0.05). Partial correlations remained significant after controlling for age, sex, VO_2_max, and delta HRV regressor (all *p*< 0.05). Changes in self-reported relaxation between conditions were not correlated with changes in HRV between conditions or with changes in HRV from the beginning to the end of the relaxation sessions (all *p* > 0.05).

## DISCUSSION

### OVERVIEW OF HEART RATE VARIABILITY FINDINGS

The present study investigated the effectiveness of theta-frequency binaural beats in enhancing the post-exercise relaxation response, quantified as trophotropic modulation of autonomic nervous system dynamics (i.e., HRV), and perceived relaxation. We hypothesized that binaural beats would increase HF power and decrease LF power and LF/HF ratio in a manner consistent with the binaural-beat entrainment model (i.e., that theta-frequency entrainment would effect trophotropic changes in autonomic arousal).

Consistent with our hypotheses, results demonstrate that, relative to placebo, 20-min of exposure to binaural beats significantly increased HF power – a known marker of parasympathetic activation that is driven by activity in regions of the anterior cingulate and medial prefrontal cortex (Wager et al., 2009). Binaural-beat exposure also decreased LF power and LF/HF ratio. Initial response to binaural beats (i.e., within the first 2-min) was characterized by significantly reduced LF power relative to placebo, with no differences in HF power or LF/HF ratio. This was the only time point where subjects showed a significant difference in mean HRV between conditions. However, significant interactions indicated a differential response to binaural beats vs. placebo, with subjects exhibiting an increase in parasympathetic dominance while listening to binaural beats, but an increase in sympathetic dominance while listening to placebo. Subjects exhibited a decrease in LF power over the first 10-min of listening to placebo but showed no change during the same period while listening to binaural beats. These findings suggest that theta-frequency binaural beats initially modulate LF power post-exercise, followed by subsequent modulation of HF power, with an overall net effect of increasing parasympathetic dominance. It appears that even a brief administration (e.g., <2-min) of binaural beats may produce acute effects on autonomic nervous system response post-exercise.

Notably, subjects entered into the relaxation protocol with a mean post-exercise heart rate of 98 bpm, which did not vary significantly by condition. After 2-min of the relaxation protocol, mean heart rate had dropped to 85 bpm, which also did not differ by condition. Research has shown that post-exercise heart rate recovery is initially mediated by a combination of sympathetic withdrawal and parasympathetic activation, but as heart rate falls below 100 bpm, parasympathetic activation begins to dominate (Pierpont and Voth, 2004). Over the course of the relaxation period, mean heart rate dropped to 74 bpm. This suggests that initial decreases in heart rate may have resulted from a combination of sympathetic and parasympathetic modulation, while subsequent decreases were increasingly mediated by parasympathetic activation (i.e., increases in HF power as well as decreased LF and LF/HF ratio).

Somewhat unexpectedly, aerobic fitness level was found to significantly interact with both Time and Condition, producing differential LF and LF/HF responses to binaural-beat vs. placebo. Specifically, in the placebo condition, greater aerobic fitness was associated with larger change scores for LF and LF/HF – suggesting that fitter individuals exhibited less sympathetic withdrawal over the course of the placebo relaxation period. Conversely, while in the binaural-beat condition, aerobic fitness was negatively correlated with relaxation LF and LF/HF change scores, suggesting that fitter individuals exhibited greater sympathetic withdrawal while listening to binaural beats. In part, this explains why simple main effects of Time were observed for the binaural-beat condition but not for the placebo condition, when aerobic fitness was included as a covariate.

### PERCEIVED RELAXATION FINDINGS

Interestingly, subjects’ sympathovagal balance during the middle of the relaxation protocol significantly predicted 43% of the variance in self-reported relaxation while listening to binaural beats, but none of the variance during the placebo condition. Further, subjects also reported being significantly more relaxed while listening to binaural beats then while listening to the placebo. Findings from this double-blind placebo-controlled study may suggest a role for binaural beats in facilitating access to more restorative states of post-exercise relaxation with subtle, yet somewhat durable psychophysiological effects. These findings should be interpreted with caution, however, given that the change in HRV measures during relaxation were not correlated with the change in relaxation scores. Regardless, results may suggest that binaural-beat associated HRV may be coupled with subjective perceptions of relaxation more so than HRV associated with standard music perception.

### LIMITATIONS AND FUTURE DIRECTIONS

In summary, we present preliminary evidence for a role of binaural beats in acutely modulating autonomic arousal, as measured through HRV. We also provide evidence linking autonomic correlates of binaural-beat exposure with a subsequent behavioral measure – perceived relaxation. Given that our sample comprised young, healthy college students, it is important for future research to investigate how binaural beats might interact with autonomic activity in a broader demographic (e.g., high-stress and clinical populations, meditation practitioners). Future studies, outside of the exercise context, that include measures of control for baseline differences in autonomic tone are warranted in order to evaluate the putative beneficial effects of theta-frequency binaural beats on the facilitation of relaxation. Further, binaural-beat technology is often designed as a training device: to assist users in accessing various altered states of consciousness. Future research will need to ascertain the extent to which structured binaural-beat training might provide for a cumulative training effect.

Although these findings suggest that theta-frequency binaural beats may facilitate relaxation post-exercise, it is important to note that other factors may have contributed to these findings. First, no measure of relaxation was taken prior to the relaxation protocol; thus, it is uncertain whether or not the observed results were a direct result of binaural-beat exposure or some other factor affecting perceived relaxation. Second, at baseline, subjects exhibited significantly lower sympathovagal balance during the binaural-beat condition relative to the control condition. It is possible that this caused the timeline of the exercise-recovery period to be offset, potentially explaining the differential autonomic response observed during the relaxation protocol between conditions. However, baseline HRV differences were controlled for in our analyses, and as previously noted, baseline sympathovagal balance predicted mid-relaxation HRV, but did not predict self-reported perceived relaxation. Only HRV indices while listening to binaural beats predicted subsequent reported relaxation. Furthermore, exercise served to bring subjects into a comparable state of physiological arousal which did not significantly differ by condition. Third, the study’s small sample size is an important limitation, although we attempted to offset this through our repeated-measures design. Future studies should attempt to replicate these findings with a sample larger than 30. Fourth, according to the BMI scale, our sample was, on average, classified as slightly overweight. It is important to note that BMI was not a primary independent variable, dependent variable, or covariate in our study. Height and weight were necessarily collected as they were used in the regression formula for predicted VO_2_max. Further, it is also important to mention that many of our study’s participants were collegiate athletes with elevated lean muscle mass, a demographic that is notoriously misclassified as ‘overweight’ or ‘obese’ by the BMI (Ode et al., 2007). Lastly, given that subjects showed an initial decrease in LF power while listening to the placebo, it is unclear to what extent the observed differences in LF power, and absence of placebo-associated changes in autonomic tone over the course of the relaxation protocol, are a result of binaural-beat assisted sympathetic withdrawal, or placebo-associated inhibition of sympathetic withdrawal. Further research with a ‘no-music’ condition will be needed to resolve this issue.

Crucially, however, the primary aim of this study was to evaluate the putative role of binaural beats in affecting autonomic nervous system activity in isolation from common confounds such as verbal guidance or instrumental music. Therefore, while the current study is not without limitations, when taking these limitations into consideration in our analytic strategy, we still demonstrated acute binaural-beat effects on parasympathetic activation and sympathetic withdrawal post-exercise.

## CONCLUSION

Acute exposure to theta-frequency binaural beats in a young, healthy sample of college students resulted in increased parasympathetic activation, increased sympathetic withdrawal, and increased self-reported relaxation post-exercise. Binaural-beat-associated HRV appeared to be more tightly coupled with self-reported relaxation than placebo-associated HRV. These findings support the putative clinical effectiveness of binaural beats in their own right, the effects of which may be synergistically enhanced through combination with other therapeutic factors such as verbal guidance and music.

## AUTHOR CONTRIBUTIONS

Patrick A. McConnell, Gary A. Sforzo and Jeffrey C. Ives were responsible for the study concept and design, and provided critical revision of the manuscript. Brett Froeliger and Eric L. Garland assisted with developing the data analysis strategy, interpreting findings, and provided critical revision of the manuscript. Patrick A. McConnell was responsible for all data collection, data analysis, drafting the manuscript, and interpreting the findings. All authors have made active contributions, critically reviewed content, and approved of the final version.

## Conflict of Interest Statement

The authors declare that the research was conducted in the absence of any commercial or financial relationships that could be construed as a potential conflict of interest.
